# The Cytidine Analog Fluorocyclopentenylcytosine (RX-3117) Is Activated by Uridine-Cytidine Kinase 2

**DOI:** 10.1371/journal.pone.0162901

**Published:** 2016-09-09

**Authors:** Dzjemma Sarkisjan, Joris R. Julsing, Kees Smid, Daniël de Klerk, André B. P. van Kuilenburg, Rutger Meinsma, Young B. Lee, Deog J. Kim, Godefridus J. Peters

**Affiliations:** 1 Department of Medical Oncology, VU University Medical Center, Amsterdam, Netherlands; 2 Department of Clinical Chemistry, laboratory Genetic Metabolic Diseases, Academic Medical Centre, Amsterdam, Netherlands; 3 Rexahn Pharmaceuticals, Inc., Rockville, Maryland, United States of America; Emory University Winship Cancer Institute, UNITED STATES

## Abstract

Fluorocyclopentenylcytosine (RX-3117) is an orally available cytidine analog, currently in Phase I clinical trial. RX-3117 has promising antitumor activity in various human tumor xenografts including gemcitabine resistant tumors. RX-3117 is activated by uridine-cytidine kinase (UCK). Since UCK exists in two forms, UCK1 and UCK2, we investigated which form is responsible for RX-3117 phosphorylation. For that purpose we transfected A549 and SW1573 cell lines with UCK-siRNAs. Transfection of UCK1-siRNA efficiently downregulated UCK1-mRNA, but not UCK2-mRNA expression, and did not affect sensitivity to RX-3117. However, transfection of UCK2-siRNA completely downregulated UCK2-mRNA and protein and protected both A549 and SW1573 against RX-3117. UCK enzyme activity in two panels of tumor cell lines and xenograft cells correlated only with UCK2-mRNA expression (r = 0.803 and 0.915, respectively), but not with UCK1-mRNA. Moreover, accumulation of RX-3117 nucleotides correlated with UCK2 expression. In conclusion, RX-3117 is activated by UCK2 which may be used to select patients potentially sensitive to RX-3117.

## Introduction

Nucleoside analogs are synthetic, chemically modified nucleosides that due to their resemblance can be incorporated into RNA and DNA to inhibit their synthesis and subsequently inhibit cell division [1]. This has potential therapeutic benefits such as the inhibition of cancer cell growth and combatting viral infections [2]. Cytidine analogs, a subclass of nucleoside analogs that are inserted into RNA and DNA replacing cytidine, are used to treat a wide variety of cancer types. Examples of successful cytidine analogs in anti-cancer applications are cytarabine and gemcitabine [2, 3], the latter drug is predominantly used for treatment of patients with non-small cell lung cancer (NSCLC) [4]. Nevertheless, the inter- and intra-tumor heterogeneity can imply for resistance to drugs in patients. Therefore, there is a need for novel anti-cancer drugs which vary in their mechanism of cellular action and thus can overcome the resistance.

**Fig 1:**
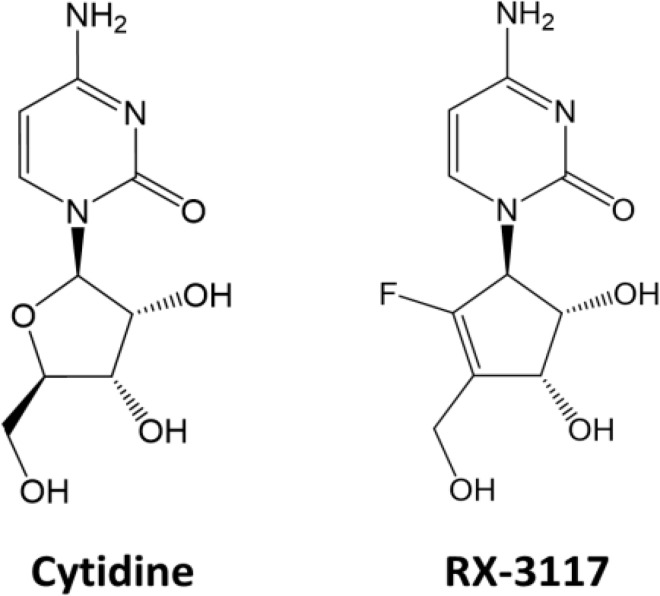
Chemical structure of cytidine and RX-3117.

A cytidine analog, fluorocyclopentenylcytosine (RX-3117) (Fig 1), has shown promise as an anti-cancer drug since it showed considerable anti-tumor activity in various xenograft models [5], including models resistant to gemcitabine [6]. The lack of cross resistance between these two drugs suggests a difference in mechanism of action or method by which they are metabolized in cells. Elucidation of the mechanisms by which RX-3117 is metabolized and exerts its cytotoxic activity is crucial in determining its strengths in a clinical setting.

A previous study provided preliminary information on its mechanism of action and metabolism [3]. Uptake of RX-3117 was shown to be mediated by human equilibrative nucleoside transporter 1 (hENT1) and its cytotoxic activity was exerted via its phosphorylated metabolites. This phosphorylation is performed by uridine-cytidine kinases (UCKs). Furthermore, this study showed that RX-3117, contrary to a drug such as gemcitabine, is not deaminated by cytidine deaminase (CDA) and that RX-3117 causes both inhibition of DNA and RNA synthesis, although the inhibition of the former is more pronounced. RX-3117 also targets DNA methyltransferase (DNMT) [3, 5] of which there are multiple variants [7]. DNMT3a and DNMT3b establish *de novo* DNA methylation patterns in DNA, which is important during embryogenesis [7], while DNMT1 differs in that its role is to maintain the established DNA methylation pattern through cell division and thus DNA replication [8]. In two previous studies a decrease in DNMT1 expression was found in cell lines treated with RX-3117 [5], while this was not the case for DNMT3a. This suggests RX-3117 might be an effective demethylating agent, comparable to decitabine (Aza-CdR) and azacytidine (Aza-CR) [1].

In order to exert its anti-cancer function, the ribonucleoside analog RX-3117 has to be phosphorylated sequentially to its monophosphate, diphosphate and triphosphate form. But it was unclear which kinase phosphorylates RX-3117 to its monophosphate form. In an effort to further elucidate the mechanism by which RX-3117 is metabolized, the current study aimed to determine which UCK is responsible for the phosphorylation of RX-3117 into its active metabolites. There are two known UCK family members: UCK1 and UCK2 [9]. UCK1 consists of 277 amino acids and is known to be ubiquitously expressed in human tissues. UCK2 on the other hand is expressed in various tumors [10, 11] and in normal human tissues it is expressed in Placenta [9]. Alternative spliced products of UCK2 gene are canonical isoforms of UCK2 which is 261 amino acids long and isoform two which is missing the C-terminal amino acids 1–150 [9]. To reveal the kinase responsible for RX-3117 phosphorylation, RNA interference (RNAi) experiments were performed targeting UCK1 and UCK2 mRNA with short interfering RNAs (siRNAs) in combination with chemosensitivity assays to assess whether UCK1 and UCK2 down regulation at RNA level had an effect on the sensitivity of lung cancer cells to RX-3117. Next we determined the enzyme activity of UCK in a panel of cell lines and xenograft cells and related this to UCK1 and UCK2 expression.

## Materials & Methods

### Cell culture

Solid tumor cell lines, A549 and SW1573 (NSCLC) and A2780 and AG6000 (Ovarian carcinoma), were cultured in Dulbecco's Modified Eagle Medium (DMEM) (Lonza, Verviers, Belgium) supplemented with 10% fetal bovine serum and 20 mM HEPES in T-25 flasks (Greiner Bio-One, Alphen aan de Rijn, the Netherlands). Non-solid tumor cell lines CCRF-CEM (Lymphoblastic leukemia) and U937 (Leukemic monocyte lymphoma) were cultured in RPMI1640 medium (Lonza) supplemented with 10% fetal bovine serum and 20 mM HEPES. The sources of the cell lines have been described earlier [3]. Cells were maintained in an experimental growth phase for all experiments and were tested negative for mycoplasma.

### Xenograft cells

Cells from various human xenografts (LS174T, BT474, RT112, Raji B, SW780, PANC-1, C-33A) were frozen in liquid nitrogen. Whole cell lysates were then prepared as described below to be used for enzyme assays, while RNA was isolated to determine expression of UCK1 and UCK2. These cells were obtained from Charles River Discovery Research Services (Seattle, WA, USA) out their cell line and tumor repository, as described earlier [9]. Xenografts were handled according to the Guide for Care and Use of Laboratory Animals, while the program is accredited by the Association for Assessment and Accreditation of Laboratory Animal Care International.

### SRB chemosensitivity assay

The sulforhodamine B (SRB) chemosensitivity assay was performed as described earlier [12] in 96-well flat-bottom plates (Greiner Bio-One). Briefly, after plating in DMEM, cells were given 24 hr to reattach to the plates and resume growth. Hereafter, the appropriate drug was added to the wells. Next, the cells were incubated at 37°C for 48 hr. Cells were then fixed with cold trichloroacetic acid (6% final concentration; Merck, Darmstadt, Germany), and incubated for at least 1 hr at 4°C. Subsequently, cells were washed with water and dried at room temperature. Fixed cells were stained with SRB dissolved in 1% acetic acid for 15 min at room temperature followed by a 1% acetic acid (Merck) wash. 10 mM Tris (Merck) base solution was added to the wells and optical density (OD) of the SRB staining was measured with a Tecan4 plate reader (Tecan, Männedorf, Switzerland) at 540 nm.

### Isolation of RNA and preparation of cDNA

RNA isolation and cDNA synthesis were performed as described earlier [13]. Briefly, cells were washed and treated with TRIzol (Life Technologies, Bleiswijk, the Netherlands) after which chloroform was added to the cell lysates and subsequently shaken and left at room temperature for 2–3 minutes. Tubes were spun at 12,000g at 4°C. The resulting upper aqueous phases containing the RNA were then transferred to fresh 1.5 ml tubes and 500 μl isopropanol was added. The tubes were mixed by inverting and incubated at room temperature for 15 minutes. The tubes were then spun at 7,500g and 4°C and washed with ice cold ethanol after which the pellets were air dried and 30 μl of nuclease free water was added. The samples were incubated for at least 30 minutes at 4°C. The tubes were flicked and spun down after which the yield was determined by using a Nanodrop ND-1000 (Thermo Fisher Scientific, Wilmington, Delaware, USA). The RNA samples were stored at -80°C.

For the synthesis of cDNA from an RNA sample, a mix was prepared consisting of reverse transcriptase buffer, random hexamer primers and reverse transcriptase (DyNAmo cDNA Synthesis kit; Thermo Fisher Scientific, Landsmeer, the Netherlands). This was thoroughly mixed with 1 μg of RNA. The tubes were placed in a thermo block with the following program: Primer extension at 25°C for 10 minutes, cDNA synthesis at 37°C for 30 minutes and reaction termination at 85°C for 5 minutes. The resulting cDNA samples were diluted 10-fold by addition of nuclease free water. Samples were then put on ice for 5 minutes. cDNA samples were stored at -20°C.

### Transfection with siRNAs against UCK1 and UCK2

To determine whether RX-3117 is phosphorylated by either UCK1 or UCK2, RNAi experiments were performed with siRNAs to downregulate their expression in cell lines A549 and SW1573. No antibiotics (penicillin and streptomycin) were used in these experiments due to a potentially negative impact on siRNA transfection of the cells. This was performed together with an SRB chemosensitivity assay to observe if their downregulation attenuated the effect of RX-3117 on the cells. Quantitative reverse transcriptase PCR (qRT-PCR) was performed to confirm successful siRNA transfection and subsequent lowered mRNA expression. The following siRNAs were used: ON-TARGETplus Human UCK1 siRNA SMARTpool, ON-TARGETplus Human UCK2 siRNA SMARTpool, ON-TARGETplus Non-targeting Pool (GE Dharmacon, Lafayette, Colorado, USA) (siNeg is not known to affect any gene expression or cell viability). The siRNAs were diluted to 5 μM according to the manufacturer’s specifications with 5x siRNA Buffer (GE Dharmacon).

Cells were transfected in 96-well flat-bottom plates (1,000 cells in 80 μl DMEM medium) (Greiner Bio-One) for subsequent SRB growth inhibition assay and in 6-well flat-bottom plates (30,000 cells in 2,400 μl DMEM medium) for subsequent qRT-PCR, western blot and immunocytochemistry. The cells were given 24 hr to reattach to the plates after which mixes of siRNAs with dharmaFECT 1 transfection reagent (GE Dharmacon) were added to the plates as described earlier [14], 20 μl for 96-well and 600 μl for 6-well plates. The mixes required for transfection of 1,000 cells in 80 μl of medium consisted of 19.45 μl serum free medium, 0.5 μl of 5 μM siRNA and 0.05 μl of transfection reagent and were prepared as specified by the manufacturer. The effective siRNA concentration during transfection was 25 nM. Fixed concentrations of RX-3117 or 3'-ethynylcytidine (ECyd) a drug known to require UCK2 for activation [15], were added 24 hr after the addition of the transfection mixes to the 96-well plates for the subsequent SRB assay. For the RNA isolation, medium was removed from the 6-well plates 72 hr post-transfection, cells were washed and lysed with 1 ml of TRIzol per well and transferred to 1.5 ml tubes. RNA was then isolated and cDNA subsequently synthesized as described previously. Next, qRT-PCR was performed with cDNA samples to determine the mRNA expression levels of UCK1 and UCK2. The following primer sets [16] were used: Forward (F), Reverse (R) and Primer (P).

UCK1 (Taqman) F: GCCGACAAAGAAGTATGCCG

UCK1 (Taqman) R: GCCATTTGCAGATGTCACCAUCK1 (Taqman) P: TGCCATCAACCTGATGTGCAGC

Primer mix for UCK1: 4 μl 200 μM F + 4 μl 200 μM R + 2 μl 200 μM P + 190 μl nuclease free water.

UCK2 (Taqman) F: GTGATCATCCCTAGAGGTGCAGATA

UCK2 (Taqman) R: GGCCCT CCATTCAGGATGT

UCK2 (Taqman) P: TCTGGTGGCCATCAACCTCATCGTG

Primer mix for UCK2: 4 μl 200 μM F + 4 μl 200 μM R + 2 μl 200 μM P + 190 μl nuclease free water.

ß-actin (Taqman) F: TCACCCACACTGTGCCCATCTACGA

ß-actin (Taqman) R: CAGCGGAACCGCTCATTGCCAATGG

ß-actin (Taqman) P: ATG CCCTCCCCCATGCCATCCTGCGT

Primer mix for ß-actin: 10 μl 200 μM F + 10 μl 200 μM R + 10 μl 200 μM P + 170 μl nuclease free water.

For each of the three primers a mix was prepared containing 14 μl MQ water, 4 μl Master^plus^ HybProbe (Roche, Almere, the Netherlands) and 1 μl of the appropriate primer per reaction. These mixes were combined with 1 μl cDNA samples and inserted in a Lightcycler 2.0 (Roche) for accurate quantification and characterization of the nucleic acids. Each qRT-PCR reaction consisted of 45 amplification cycles.

### Immunocytochemistry and immunohistochemistry

For visualization purposes, cells were fixed with 4% paraformaldehyde solution on StarFrost microscope slides (Knittel Glass, Germany) for 15 minutes. Freshly frozen tissue from patients was handled as described previously [17]. The samples were obtained from our tumor Biobank as described earlier [17] and its use for scientific purposes was allowed by the ethical committee. Endogenous peroxidase activity was blocked during 20 minutes incubation with 0.3% H_2_O_2_ (VWR Prolabo, #23622–260) solution. The blocking step was performed using 5% BSA (Sigma-Aldrich, #A3294) with addition of 0.1% Triton X-100 (Thermo Scientific, #28314) solution. The staining was carried out using primary polyclonal rabbit antibody UCK2 (1:200, YK-582, Abcam, Burlingame, CA) overnight, 30 minutes with anti-rabbit biotinylated (1:300, Vector biotin, BA-1000), following 30 minutes incubation with HRP-streptavidin (1:300, DAKO Cytomation, PO397). To visualize the UCK2 antibody, 3,3′-diaminobenzidine (DAB) (10 μg/ml, Sigma, #D5637) staining was performed with addition of 0.3% H_2_O_2_ prior to use.

### UCK2 protein expression analysis

The UCK2 protein expression and downregulation of UCK2 protein expression with RNAi against UCK2 in SW1573 and A549 cell lines were analyzed by western blots essentially as described earlier [18]. Briefly, cells were lysed using cell lysis buffer (Cell Signaling, Danvers, MA, USA) containing 4% protease inhibitor cocktail (Roche Diagnostics, Mannheim, Germany) on ice for 30 minutes and centrifuged for 10 minutes at 4°C at 14,000 rpm. Bio-Rad assay was performed to determine protein amount in the supernatant as described earlier [18]. The following antibodies were used: UCK2 (1: 1000, YK-582) and β-actin (1: 10,000, Sigma, St. Louis, USA). Additional antibodies against UCK1 and UCK2 were prepared as described earlier [19]. Antibodies were diluted in a mixture of Rockland buffer (Rockland Inc, Philadelphia, PA) and phosphate-buffered saline (PBS) supplemented with 0.05% Tween 20. Proteins were separated in 20% SDS-PAGE and transferred to polyvinylidene difluoride (PVDF) membrane. For fluorescent signal secondary anti-bodies goat anti-mouse InfraRedDye and goat anti-rabbit InfraRedDye were used. Proteins were detected by an Odyssey InfraRed Imager (Li-COR Bioscience, Lincoln, NE).

### Correlation of UCK enzyme activity with UCK1 and UCK2 expression and RX-3117 metabolism

In order to determine whether UCK2 expression can potentially be used as a predictive biomarker in tumors, we analyzed the potential correlation with UCK enzyme activity. UCK enzyme activities were determined with radioactively labeled [^3^H]-RX-3117 and [2-^14^C]-uridine as described earlier [3] in a panel of six tumor cell lines and seven xenograft cells. Expression data of UCK1 and UCK2 were determined after RNA isolation, reverse transcription and qRT-PCR as described above.

In order to measure intracellular phosphorylated RX-3117, we exposed intact cells to 10 μM [6-^3^H]-RX-3117 (90.5 mCi/mmol) for 120 minutes as described earlier [3]. The reaction was stopped by acid precipitation RX-3117 and its metabolites were analyzed using layer chromatography as described earlier [3].

### Cell cycle and proliferation analysis

The A549 and SW1573 cells were seeded in 12 well plates (Greiner Bio-One GmbH, Frickenhausen, Germany) at the density of 10^5^ cells and allowed to attach for 24 hr. After 24 hr the treatment was started by adding 33.3 μM RX-3117 or 33.3 μM RX-3117 plus 100 μM of uridine or cytidine. For the proliferation analysis, cells were counted at the start of the treatment using a Coulter counter and after 24 hr of treatment cells were counted again and doubling time was calculated using Roth V. 2006 Doubling Time Computing, available from: http://www.doubling-time.com/compute.php. Cell cycle distribution was determined in the total amount of adherent and floating cells that were collected in round-bottom FALCON tubes (BD, Franklin Lakes, NJ, USA). After centrifugation, cell pellets were resuspended in 0.5 ml propidium iodide (PI) solution (50 ug/ml PI, 0.1% sodium citrate 0.1% Triton X-100, 0.1 mg/ml ribonuclease A) and left on ice for 30 minutes. Subsequently, samples were analyzed using FACSCalibur (BD Biosciences, Mount View, CA, USA). For data analysis CELLQuest ™ software was used by setting gates on DNA histograms to estimate the amount of cells in different phases.

### Analysis and statistics

When applicable the Student’s t-test was performed to determine whether differences were significant. Pearson correlation analysis was used to determine strengths of relations. The cut-off value for significance was set at p<0.05.

## Results

### RX-3117 activation

**Fig 2:**
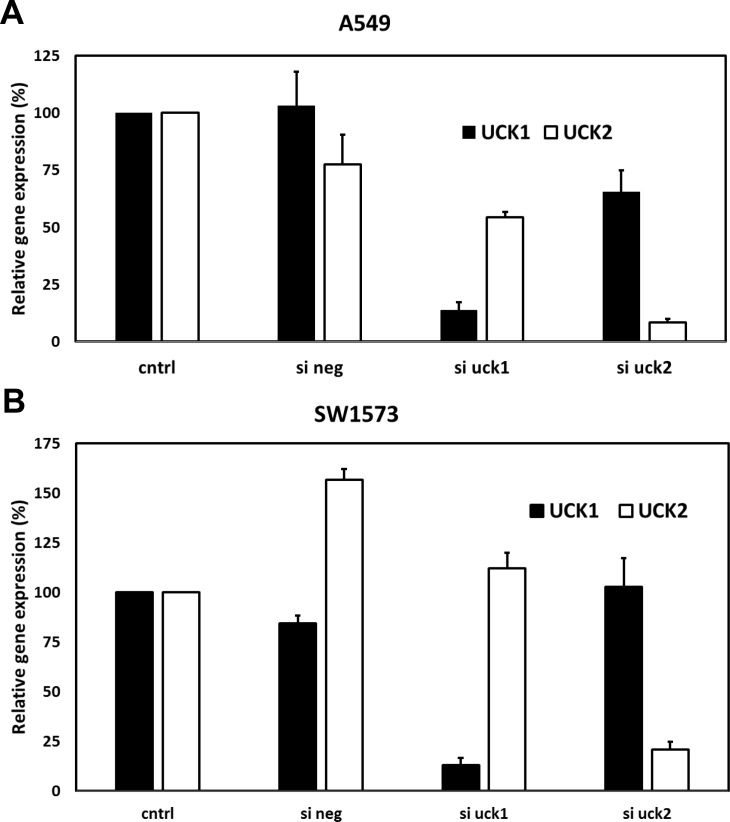
UCK1 and UCK2 mRNA levels 72 hr after siRNA transfection. **(A)** Relative UCK1 and UCK2 mRNA levels after transfection in A549 cells. **(B)** Relative UCK1 and UCK2 mRNA levels after transfection in SW1573 cells. Values are means ± SEM from 2–3 separate experiments. The gene expression in control cells was set at 100% for each separate experiment in order to calculate the relative gene expression in siRNA treated cells.

**Fig 3:**
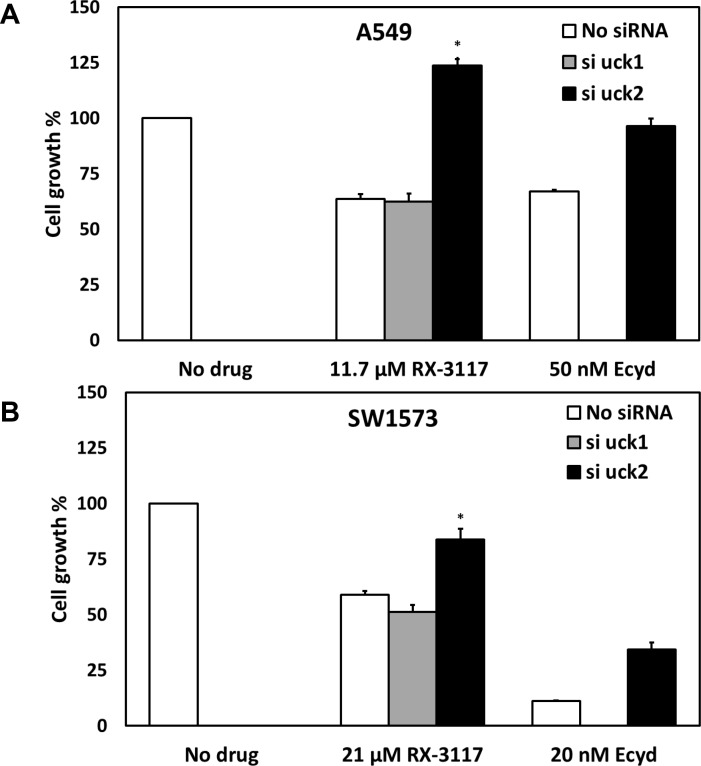
**Effect of UCK1 and UCK2 down regulation on RX-3117 cytotoxicity in A549 (A) and SW1573 (B) cells.** Values are means ± SEM (n = 6). Values were normalized to 100% for untreated cells. As controls we also used cells transfected with control scrambled siRNA (data not shown). Drugs were added 24 hr after transfection. Cells were exposed to drugs for 48 hr. ECyd was used as a positive control. The protective effect of siUCK2 was significant in both A549 (p = 0.004) and SW1573 cells (p = 0.003). Significance is marked with an asterisk (*).

RNAi experiments were performed using siRNAs targeting UCK1 and UCK2 to determine which of the two kinases was responsible for phosphorylation of RX-3117. In A549 cells the siRNA transfection resulted in proper downregulation of UCK1 (90% reduction) and UCK2 (93% reduction) at the mRNA level, although siUCK2 seemed to have a small off-target effect on UCK1 expression (Fig 2A). Expression levels were normalized to the level of expression of ß-actin. siRNA downregulation in the SW1573 cell line yielded similar results, showing an 87% reduction of UCK1 and 79% reduction of UCK2 mRNA, although the siRNA negative sample showed an increase in UCK2 expression (Fig 2B). Transfection of the cells with siRNA against UCK1 or UCK2 resulted in decreased viability. For the chemosensitivity experiments as shown in Fig 3 these values were normalized to 100%.

For both siRNA transfected cell lines a chemosensitivity assay was performed with RX-3117 to determine whether downregulation of either UCK1 or UCK2 would reduce the efficacy of RX-3117 (Fig 3A and 3B). ECyd was used as a positive control. A549 cells were moderately sensitive to 11.7 μM RX-3117 (63.7% cell growth), but the cells transfected with siUCK2 were completely protected (123.6% cell growth), while this was not the case in cells treated with scrambled siRNA negative control (63.7% cell growth; not shown) or siUCK1 (62.4% cell growth). The difference between control and siUCK2 was statistically significant (p = 0.004, n = 6). Results of cells treated with ECyd were the same, with complete recovery (96.5% cell growth) from the cytostatic effect of the drug (67% cell growth) (Fig 3A). In SW1573 cells siUCK2 also reduced (83.8% cell growth) the cytostatic effect of 21 μM RX-3117 (59% cell growth; p = 0.003, n = 6). There was no protection from its cytostatic effect in cells treated with scrambled siRNA negative control (50.6%; not shown) or no siRNA (59.0% cell growth) and siUCK1 (51.4% cell growth). The ECyd negative control also showed a reduction (34.4% cell growth) in the cytostatic effect of the drug (11.2% cell growth) in cells transfected with siUCK2 (Fig 3B).

### Efficiency of UCK2 down regulation and UCK2 expression in tumor and normal tissues

**Fig 4:**
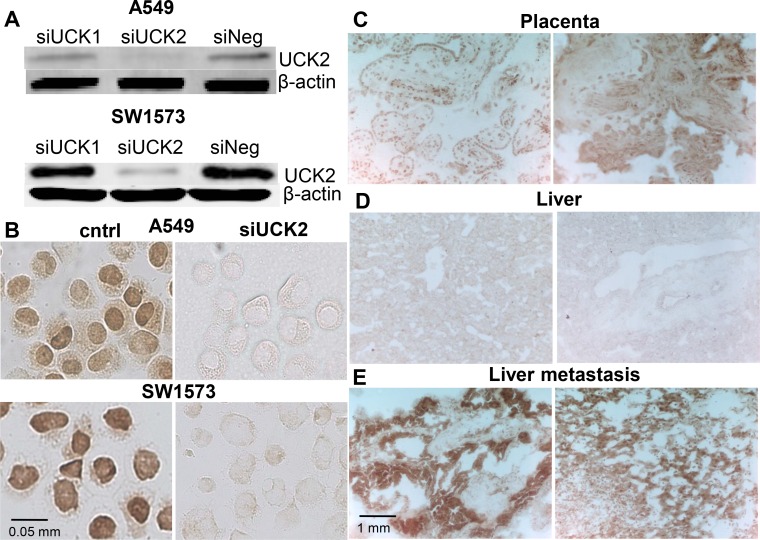
Validation of UCK2 down regulation and UCK2 expression in normal and cancer tissues. Silencing of UCK2 measured by western blotting; expression in cells treated with siRNA against UCK1 and UCK2 was compared to no siRNA (siNeg) **(A)**. For immunocytochemistry; silence of UCK2 by siRNA was compared to untreated control in A59 and SW1573 **(B)**. Examples of normal placenta from two women stained with the UCK2 antibody **(C)**, normal liver from two patients **(D)** and liver metastases from separate human colorectal cancers **(E).**

Next, the capability of siRNA directed to a specific gene product sequence to reduce UCK2 protein expression was examined. The two cell lines A549 and SW1573 were transfected with siUCK1 and siUCK2 to be analyzed by western blotting and immunocytochemistry. In both cell lines siUCK2 transfection resulted in an almost complete downregulation of UCK2 protein, while siUCK1 transfected cells showed a high UCK2 expression comparable to scrambled siRNA transfected ones (Fig 4A). To further validate UCK2 silencing, immunocytochemistry was performed. The majority of A549 and SW1573 cells exhibited positive or highly positive UCK2 staining (Fig 4B). Moreover, UCK2 seemed to have a partial nuclear localization (Fig 4B). SW1573 cell line showed a higher UCK2 staining compared to A549 cells. Both cell lines showed an efficient UCK2 protein down regulation upon siUCK2 transfection (Fig 4B).

In order to verify the specificity of UCK2 staining we stained tissues known to have either high or low UCK2 expression [9–11]. Human placenta indeed showed a high expression of UCK2 as well as samples from human liver metastases derived from colon cancer (Fig 4C and 4E). In normal liver obtained from the same patients we could not detect any UCK2 positive staining (Fig 4D).

### Relation between UCK activity and UCK1/UCK2 expression

**Fig 5:**
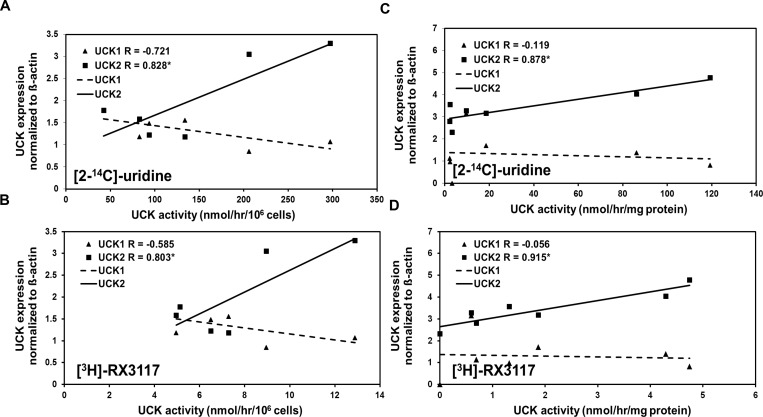
**Regression analysis of UCK1/UCK2 expression and enzyme activity for [2-**^**14**^**C]-uridine and [**^**3**^**H]-RX-3117 in the cell line panel (A and B) and xenograft cells (C and D).** In both panels UCK2 mRNA expression correlated significantly (p<0.001) with UCK enzyme activity measured with both [2-^14^C]-uridine (r = 0.828 for the cell lines and r = 0.878 for the xenograft cells) and [^3^H]-RX-3117 (r = 0.803 for the cell lines and r = 0.915 for the xenograft cells). UCK1 mRNA expression did not correlate with enzyme activity with either substrate (r = -0.721 and -0.585, respectively for the cell lines and r = -0.119 and r = -0.056 respectively for the xenograft cells). The cell lines in panel A and B shown in increasing UCK activity are: U937, A549, CCRF-CEM, SW1573, AG6000 and A2780. In panel C and D the sequence is LS174T, BT474, RT112, Raji B, SW780, PANC-1 and C-33A. The r-coefficient is given in the legend along for each gene. Significant correlations are marked with an asterisk (*).

To determine whether the expression of UCK1 or UCK2 correlates with UCK activity, their mRNA expression was measured in a panel consisting of six tumor cell lines (U937, A459, CCRF-CEM, SW1573, AG6000 and A2780) and in a panel derived from seven xenograft cells (LS174T, BT474, RT112, Raji B, SW780, PANC-1, C-33A). The UCK activity data of the 6 cell lines were previously obtained [3]. Linear regression analysis showed that UCK2 mRNA expression was significantly (p<0.001) correlated with UCK enzyme activity in both panels measured with both [2-^14^C]-uridine (r = 0.828 for the cell lines and r = 0.878 for the xenograft cells) and [^3^H]-RX-3117 (r = 0.803 for the cell lines and r = 0.915 for the xenograft cells), while this was not the case for UCK1 (r = -0.721 and -0.585, respectively for the cell lines and r = -0.119 and r = -0.056 respectively for the xenograft cells) (Fig 5A, 5B, 5C and 5D).

**Fig 6:**
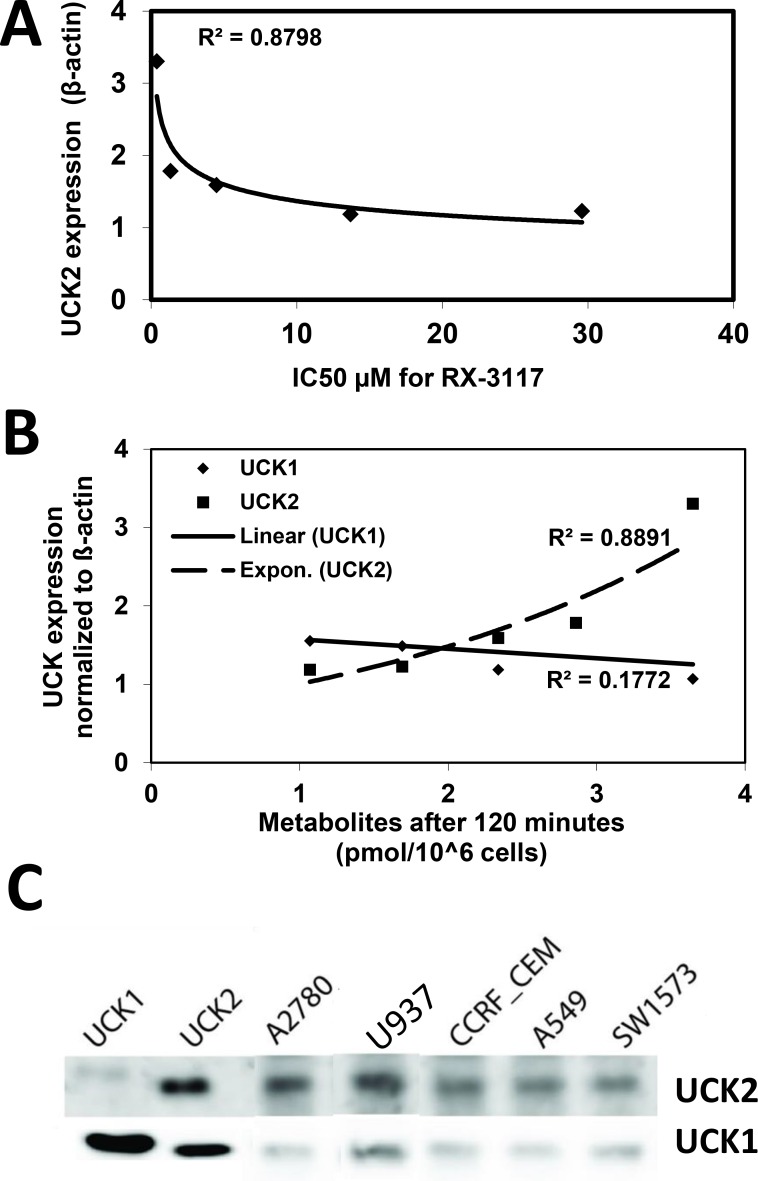
**Correlation between the UCK2 mRNA expression of the cell lines in panels A and B (Fig 5) with phosphorylation and the sensitivity to RX-3117**. The cell lines starting from highest UCK2 expression and sensitivity are: A2780, U937, A549, SW1573 and CCRF-CEM. **(A)** Correlation between sensitivity to RX-3117 and mRNA expression of UCK2. **(B)** RX-3117 phosphorylation in panel cells. RX-3117MP formation after 120 minutes correlated with UCK expression. **(C)** Protein expression of UCK1 and UCK2; the two left lanes are positive controls for UCK1 and UCK2 for which high levels of purified protein was used, which shows some cross-reactivity. Equal amounts of protein were put on the gels.

We also investigated the role of UCK2 in sensitivity to RX-3117 in the above mentioned cell lines. We observed a non-linear correlation of UCK2 mRNA expression and sensitivity to RX-3117 (Fig 6A). Cells with a similar, although relatively low, UCK2 expression showed a range in sensitivity to RX-3117. However, none of the cell lines was negative for UCK2 expression in the range of down-regulation with siUCK2. Above a certain threshold sensitivity increased.

In these cell lines we also investigated the formation of activated RX-3117 nucleotides (Fig 6B). The formation of RX-3117MP at 60 and 120 minutes, which is completely dependent on the activation by a UCK, showed an excellent correlation (0.8768 and 0.8891, respectively) with the expression of UCK2, but absolutely no correlation with that of UCK1. For the higher nucleotides, for which accumulation is dependent on more factors, the correlation was lower, for RX-3117DP the correlation coefficients were 0.7710 and 0.5634, respectively), while for RX-3117TP these values were 0.0403 and 0.0259, respectively).

Next to the mRNA expression we also measured protein expression with two newly developed antibodies [19]. Interestingly protein expression of UCK1 was low in the cells (Fig 6C), but histidine-tagged UCK1 and UCK2 controls both showed positivity when stained with UCK1 antibody, probably because of very high pure protein level that was used. The UCK2 protein expression was high in the most sensitive cells from the panel A2780 and U937 (Fig 6C). This UCK2 protein expression data apply for the earlier proposed existence of a threshold that needs to be reached for sensitivity to RX-3117 and the hypothesis that UCK activity in cells is due to UCK2 and not UCK1.

### Downstream effects of RX-3117 disappear upon competition with UCK2 substrate

**Fig 7:**
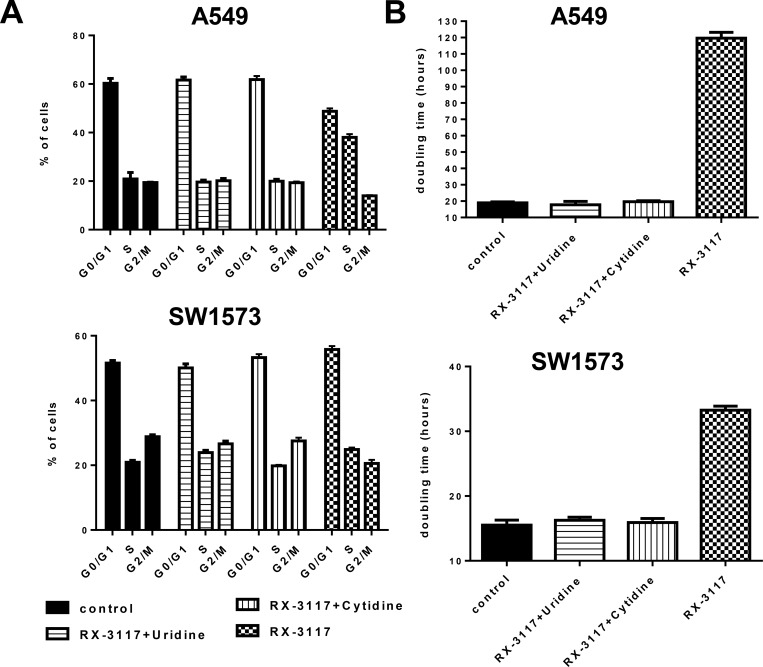
Rescue of cell cycle effects and proliferation inhibition of cell lines upon treatment with RX-3117 conditioned with Uridine or Cytidine. **(A)** The cell cycle distribution was monitored 36 hours after exposure to 33.3 μM RX-3117 or 33.3 μM RX-3117 with 100 μM of uridine or cytidine in A549 and SW1573 cells. **(B)** Proliferation of A549 and SW1573 cell lines treated with 33.3 μM of RX-3117 during 24 hours or with RX-3117 plus 100 μM of uridine/cytidine.

Earlier we showed that an excess of the UCK2 substrates uridine and cytidine would protect cells from cytotoxicity of RX-3117 by preventing that RX-3117 is activated by UCK2. As a read-out for a mechanistic effect we analyzed the cell cycle distribution of A549 and SW1573 cells after exposure to 33.3 μM RX-3117 for 24 hrs or RX-3117 with 100 μM uridine or 100 μM cytidine. PI stained cells showed an increase in the S-phase when treated with RX-3117 (Fig 7A) in A549 cells from 20.92% to 38.06% and in SW1573 cells from 20.91% to 24.87% (Fig 7A). However, both uridine and cytidine completely abolished the effect on the cell cycle, showing that UCK2 activation is essential for the cellular effects of RX-3117. The effect on cell proliferation was investigated after treatment with 33.3 μM RX-3117 for 24 hrs or RX-3117 with 100 μM uridine or 100 μM cytidine. In A549 cells doubling time changed from 19 hrs in control condition to 120 hrs when RX-3117 was administered alone. In SW1573 cells doubling time changed from 16 hrs in control condition to 33 hrs in RX-3117 treated conditions. In both cell lines addition of uridine or cytidine along with RX-3117 resulted in doubling times similar to control conditions (Fig 7B).

## Discussion

This study demonstrates that the novel cytidine analog fluorocyclopentenylcytosine (RX-3117) is activated by UCK2 to its monophosphate. UCK1 does not play a role in this process. Moreover, UCK2 expression is high in a number of target tumor tissues.

UCKs catalyze the phosphorylation of uridine and cytidine and their respective synthetic analogs [9]. UCK activity and the expression of UCK1 can be found in most tissues, but UCK2 expression in healthy human tissue has only been found in the placenta [9]. Our data on UCK2 expression in placenta and tumors obtained with a specific antibody against UCK2 are in agreement with these studies. In addition tumors have a higher activity of UCK compared to normal tissue, such as the absence of UCK2 expression in liver tissue, while tumors also show an increase in UCK2 expression, indicating that the increase is related to the expression of UCK2 [9, 16]. Both UCK1 and UCK2 have been associated with resistance and sensitivity for Aza-CR. A lower UCK1 expression for myelodysplastic syndrome (MDS) patients has been associated with reduced survival rate, while point mutations in UCK2 have been found in human leukemic cell lines made resistant to Aza-CR [20, 21]. A previous study already demonstrated that RX-3117 is activated by UCKs by competitively inhibiting UCK mediated drug activation with the natural nucleosides uridine and cytidine [3], but it remained unclear whether RX-3117 was activated specifically by UCK1 or UCK2. The use of siRNA transfection in A549 and SW1573 cell lines coupled with RX-3117 sensitivity assays showed that activation of RX-3117 is carried out by UCK2 mediated phosphorylation and that UCK2 is the single actor responsible for the first activating step of RX-3117. Similar results were obtained with ECyd as a positive control, a drug earlier shown to be solely activated by UCK2 [15]. The silencing of UCK2 was validated with different techniques. In addition, the downstream effects of RX-3117 were completely abolished by inhibition of UCK2, indicating that UCK2 is the important limiting kinase in activation of RX-3117 for its downstream effects. Both protein and gene expression of UCK1 did absolutely show no correlation with RX-3117 metabolism and sensitivity. All together the evidence is substantial to draw a conclusion.

Our findings provide novel perspectives for RX-3117, since it can circumvent resistance to other widely used drugs such as gemcitabine, commonly used for treatment of several solid tumors including lung [4] and pancreatic cancer [22]. Gemcitabine is phosphorylated into its monophosphate state by deoxycytidine kinase (DCK) [23], but UCK2 plays no role. This explains the lack of cross resistance between gemcitabine and RX-3117 and will increase the potential of RX-3117 to be used against these tumors. Along with being active in gemcitabine resistant cell lines in vitro [3, 5], RX-3117 also showed *in vivo* activity against various tumor types, including gemcitabine resistant tumors [5, 6]. Additionally, this study also showed a relation between UCK2 expression and UCK activity as well as of UCK2 expression and RX-3117 sensitivity, which suggests that UCK2 expression may be used to select patients for their sensitivity to RX-3117. Since UCK2 is low in normal tissues, it is unlikely that RX-3117 is activated in normal tissues, potentially reducing the occurrence of toxicity at therapeutic doses.

In addition to the pathway by which RX-3117 is metabolized, the mechanism of action by which it exerts its cytotoxic activity is equally important. Earlier it was shown that RX-3117 downregulates DNMT1 [3], however the mechanism of downregulation needs to be further elucidated as its structure does not contain a nitrogen in place of a carbon at position 5 of the pyrimidinone ring, which is characteristic for the major demethylating agents aza-CdR and aza-CR. They inhibit DNMT1 by forming a tight covalent bond with the enzyme after incorporation into the DNA [24].

To summarize, this study showed that UCK2, but not UCK1, is solely responsible for the phosphorylation of RX-3117 into its monophosphate metabolite. This distinctive metabolism by tumor cells compared to e.g. gemcitabine offers a potential for clinical application due to its lack of cross resistance.
